# A randomized controlled trial to explore the efficacy and safety of transcranial direct current stimulation on patients with post-stroke fatigue

**DOI:** 10.1097/MD.0000000000027504

**Published:** 2021-10-15

**Authors:** Xiang-Li Dong, Xing Sun, Wei-Ming Sun, Qin Yuan, Guo-Hua Yu, Lang Shuai, Ye-Feng Yuan

**Affiliations:** a Department of Psychosomatic Medicine, The Second Affiliated Hospital of Nanchang University, Nanchang, China; b First Clinical Medical School, Nanchang University, Nanchang, China; c Department of Rehabilitation Medicine, The First Affiliated Hospital of Nanchang University, Nanchang, China; d Department of Psychology, Jiangxi University of Traditional Chinese Medicine, Nanchang, Jiangxi Province, China; e Department of Psychosomatic Medicine, The First Affiliated Hospital of Nanchang University, Nanchang, China.

**Keywords:** fatigue, post-stroke, RCT, rehabilitation, transcranial direct current stimulation

## Abstract

**Background:**

Post-stroke fatigue seriously affects the quality of life for stroke patients. There is no effective treatment at present. transcranial direct current stimulation (tDCS) is a non-invasive brain stimulation which may have therapeutic effect on post-stroke fatigue. This study will explore about this.

**Method:**

A total of 60 patients with post-stroke fatigue were randomly divided into the control group and the treatment group with 30 patients each by minimization randomization. Both groups received basic treatment and conventional rehabilitation. In the treatment group, patients were treated with active tDCS, while in the control group, sham tDCS. Both active and sham tDCS were administered 6 times a week for 4 weeks. Before and after the trial, the Fatigue Severity Scale (FSS), Fugl-Meyer Assessment (FMA) and Modified Barthel Index (MBI) were evaluated and analyzed. And comparisons were made among groups. And there were an 8-week follow-up after the intervention.

**Result:**

Before the intervention, there were no significant differences in baseline data and assessment scores between the groups (P > 0.05). After 4 weeks of intervention, FSS scores in the treatment group were significantly lower than those in the control group (P = 0.012), and FMA and BMI scores were significantly higher than those in the control group (P < 0.05). There was no significant change in FSS scores after 8 months of follow-up (P > 0.05).

**Discussion:**

TDCS is a safe treatment that can effectively reduce the degree of fatigue after stroke, improve the motor function and daily activity ability of patients after stroke, and the efficacy is better than only routine rehabilitation training.

**Trial registration number:**

Chinese Clinical Trial Registry, ChiCTR2000031120. Registered on March 22, 2020.

## 1 Introduction

Fatigue is defined as the decrease in physical and/or mental performance that results from changes in central, psychological, and/or peripheral factors.^[1]^ Post-stroke fatigue (PSF) is defined as observable and measurable performance degradation that occurs during the repetition of a physical or mental task.^[2]^ It is an early feeling of exhaustion, boredom, and aversion to effort. PSF was first mentioned in 1999, and has been evaluated separately by the medical community to distinguish it from other post-stroke psychosocial disorders. According to previous studies, 39% to 72% of stroke survivors have PSF.^[3,4]^ Descriptions of fatigue include different aspects of the phenomenon, problems related to self-control and emotional instability, reduced mental capacity and energy requirements for daily activities, such as reading and participating in physical or social activities. Fatigue can be divided into objective fatigue and subjective fatigue.^[5]^ And PSF is often referred to as subjective fatigue. As for the characteristics of fatigue, PSF is qualitatively different from pre-stroke fatigue that may be exacerbated by stress and physical activity while responding well to rest, sleep, and hypothermia. The PSF aggravated by physical activity is also known as exercise-induced fatigue. Exercise-induced fatigue is acute, with rapid onset, short duration, and a short recovery period. It usually occurs after vigorous physical exercise or the use of mental work. PSF, on the other hand, is chronic, long lasting, and difficult to recover from. It can occur in the persistent activities of daily life such as taking a shower.

About the pathogenesis of PSF, repeated exhaustive exercise could result in upregulation of the expression of mGluR1 and mGluR5 in the cortex M1 zone of rats suggesting that M1, with different time effects, contained important receptors related with the production of exercise fatigue. A transcranial direct-current stimulation (tDCS) of M1 and dorsolateral prefrontal cortex (DLPFC) significantly increased brain excitability in the M1 for at least 30 minutes.^[6]^ Therefore, it suggests that we can improve fatigue by tDCS on the DLPFC.

tDCS is a noninvasive brain stimulation method that regulates cortical excitability by applying 1 to 2 mA direct current through the scalp. It requires at least one stimulator, electrode, and return electrode to loop. It can be divided into anode and cathode stimulation mode. By using tDCS to stimulate the motor cortex, it was found that anode stimulation can improve cortical excitability,^[7]^ whereas cathode stimulation can reduce cortical excitability. Furthermore, the effect of it is not only limited to the stimulated area, but also involves nearby brain regions^[8]^ and can change the functional connectivity between brain regions.^[9]^ If the time and intensity of stimulation are enough, the change of cortical excitability after a single stimulation can last for about 1 hour.^[10]^ In previous experiments,^[7]^ tDCS was found to improve fatigue using sham-controlled crossover designs, with between 10 and 25 participants and 5 tDCS treatment sessions using a motor, sensory, or DLPFC montage.^[11,12]^ The most recent study by Chalah et al^[12]^ demonstrated that the DLPFC (left anodal) when compared with the posterior parietal cortex led to the highest fatigue-specific improvements. One of the advantages of tDCS is that it can be regulated by the experimenter actively. Compared with transcranial magnetic stimulation, it is obvious that tDCS has a lower resolution. Conversely, it is less expensive, portable, and easier to use. More importantly, it was found that it has no side effects other than a slight tingling sensation.

Recently, tDCS is increasingly being used in many fields, such as dysphagia, head injuries, Alzheimer disease, Parkinson disease, acute and chronic pain, tinnitus, and depression. It is also being tested on healthy people.^[13]^ However, few clinical studies have reported a combination of tDCS and PSF recovery.

## 2 Materials and methods

### 2.1 Inclusion criteria

(1) Stroke appeared at least 3 months ago and within 1 year, to ensure that they are not in the acute phase of stroke.
(2) Apparent fatigue, decreased energy, need for increased rest time, or fatigue out of proportion to physical activity.
(3) One of the following is true (FSS score is >36):
  1) sleep or rest is difficult to achieve or recover,
  2) motivation is retained and productivity is reduced,
  3) self-perception is required to overcome this lack of vitality,
  4) fatigue affects daily life/tasks,
  5) fatigue lasts for several hours after exercise,
  6) fatigue is a significant concern,
(4) male or female patients aged 18 to 65 years.
(5) by magnetic resonance imaging (MRI), patients with no significant head displacement, structural damage, extensive necrosis of the brain structure, no significant pyramidal tract necrosis or thalamic injury in the brain stem, and not >30% of each lobe damaged on one side of the brain.
(6) the patient's condition and vital signs are stable, and the patient's family voluntarily participated in the trial and signed an informed consent form.
(7) the patient has no other serious complications, such as respiratory failure, acute heart failure, severe pulmonary infection, upper gastrointestinal bleeding, etc.

### 2.2 Exclusion criteria

(1) Sedatives, anesthetics, psychoactive drugs, muscle relaxants, or Na+ and Ca2+ channel blockers, such as carbamazepine, will be administered during the evaluation period.
(2) The patient relies on inhalers.
(3) The course of disease is >1 year.
(4) There are any contraindications, such as pacemaker, denture, and metal prosthesis, etc.
(5) There is an epilepsy or seizure history confirmed by electroencephalo-graph (EEG).
(6) Serious diseases such as heart, liver, and kidney failure.
(7) Progressive nervous system diseases, such as central nervous system or degenerative diseases.
(8) The patient has fever.
(9) The patient has a local skin injury or inflammation.
(10) The patient has hemostasis, coagulation, or anticoagulation dysfunction.
(11) Acute large area cerebral infarction.
(12) High sensitivity of pain stimulation area.
(13) Brain injury, brain parasitosis, or brain tumor.
(14) Hemiplegia or dysfunction of limbs (patients with a Fugl-Meyer scale score below 85).
(15) Accompanied by aphasia.
(16) Patients with incomplete clinical data and poor compliance.
(17) Accompanied with serious organ disease, endocrine disease and mental illness.
(18) Patients with a score of ≥10 on the PHQ-9 scale.

### 2.3 Method

**Figure 1:**
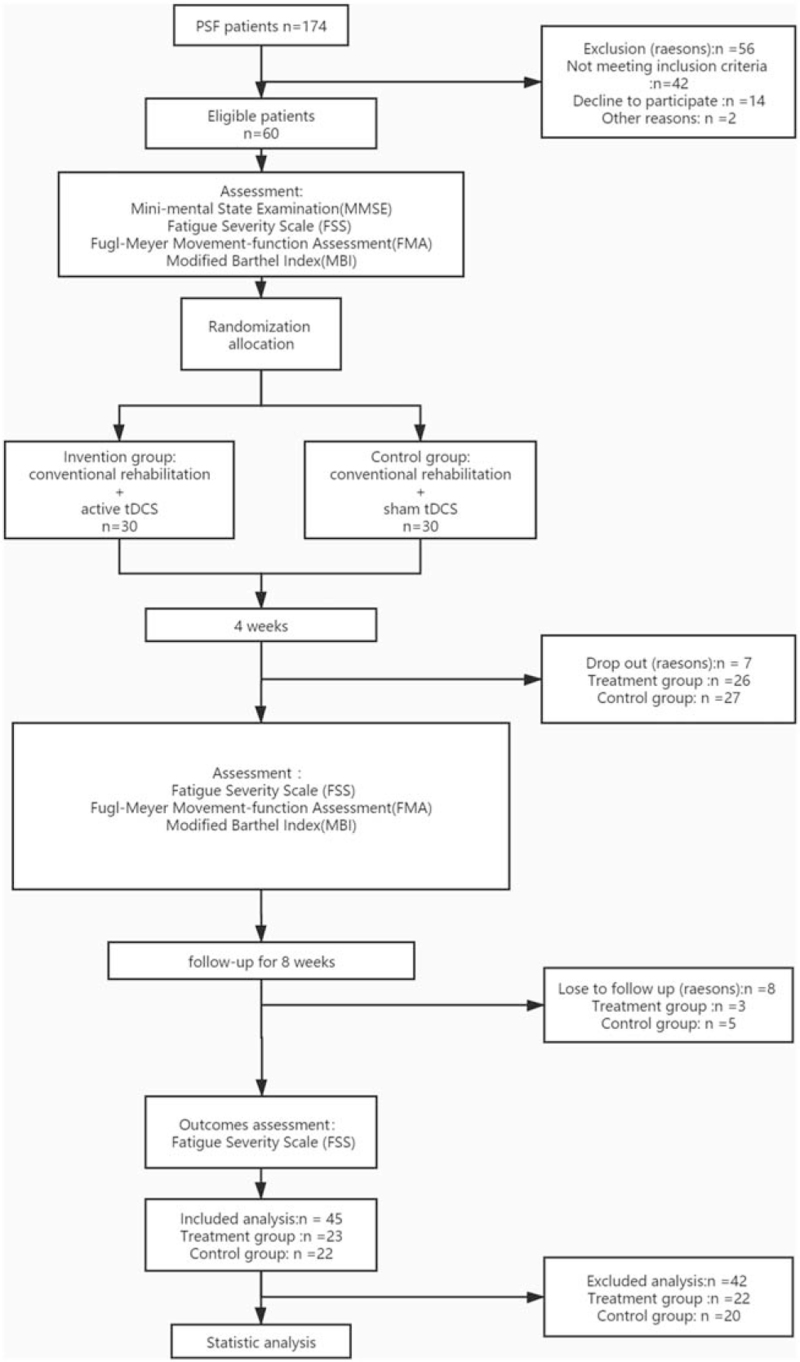
Flow diagram of the trial design. PSF = post-stroke fatigue, tDCS = transcranial direct current stimulation.

This study was carried out with 60 hospitalized patients with PSF from January 25, 2020 to July 25, 2020. All participants were randomly allocated to either the intervention or control group in a 1:1 ratio by minimization randomization. Each group was composed of 30 patients. The control group received conventional rehabilitation combined with sham tDCS treatment, whereas the treatment group received conventional rehabilitation combined with active tDCS treatment. This trial was composed of a 4-week intervention and an 8-week follow-up period. From the first to the fourth week, the primary outcome fatigue severity scale (FSS) was measured at baseline at the end of treatment. The secondary outcomes Fugl-Meyer movement-function assessment (FMA), modified Barthel index (MBI) was performed at the end of the intervention period. Adverse events and untoward effects were supervised in each treatment. The first affiliated Hospital of Nanchang University (NCU), which undertook the study, was responsible for training rehabilitation therapists on standard operating procedure and supervising the progress of this trial at all clinical sites. In addition, the randomization and blinding were performed by an independent statistician from the Center of Evidence Based Medicine, NCU (Table 1, Fig. 1).

**Table 1:**
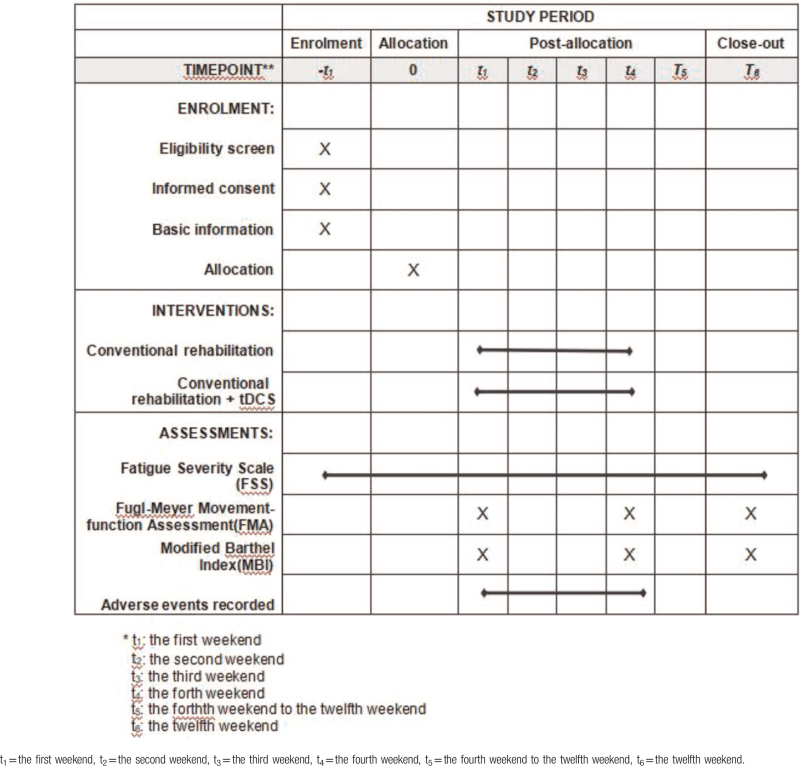
Data on the design of the study.

#### 2.3.1 Basic treatments

Basic regular treatments included controlling intracranial pressure, blood pressure, body temperature, and blood sugar of patients. Other treatments included prevention and treatment of platelet aggregation, maintenance of electrolyte and acid-base balance, prevention of complications, inchoate rehabilitation treatment for patients, regular turning over for patients, attention to the placement of limbs, daily training of joint muscles, up and down stairs, and daily life self-care ability. Depending on the patient's situation, the clinician managed each patient, including the drug use and prevention of complications.

#### 2.3.2 Active tDCS

The treatment group participants were treated with active tDCS. We placed the anode of the electrode on the DLPFC on the left side of the patients’ forehead, and cathode on the superior margin of the right orbit. The current intensity was 1.5 mA according to previous article.^[14]^ The device used is MBM-I (Nanchang City, Jiangxi Province, China). The electrode plate is a circle with a diameter of 5 cm. The treatment parameters were 20 minutes per session, once a day, and 6 times a week. Patients received tDCS treatment alone, not at the same time as other treatments. The treatment was carried out in the neuromodulation room, at the appointed time, by a specialized therapist.

#### 2.3.3 Sham tDCS

Participants in the control group were treated with sham tDCS treatment. The anode of the electrodes was located at the DLPFC on the left side of the forehead, and cathode at the superior margin of the right orbit. The current was only input every 15 seconds during the initial phase, and there will be no current output during the intermediate 19.5 minutes of sham stimulation. The rest of the parameters was the same as for active stimulation.

### 2.4 Follow up

After the 4-week treatment period, the patients were followed up for 8 weeks by phone. Participants were contacted every 2 weeks to record medication and rehabilitation. During the last week of follow-up (the 8th week after the intervention), participants were referred for clinical evaluation to assess their fatigue level by FSS.

### 2.5 Trial outcomes

#### 2.5.1 Primary outcomes

##### 2.5.1.1 Fatigue severity scale (FSS)

FSS is one of the most widely known and used scales composed of 9 items and evaluated by 7 points.^[15–21]^ FSS points increase from 1 point (highly disagree) to 7 points (highly agree). In 1989, Krupp et al^[22]^ developed the scale and applied it to patients with systemic lupus erythematosus and multiple sclerosis. Aside from multiple sclerosis (MS), this scale has also been used in Parkinson disease, chronic fatigue syndrome, brain injury, and other diseases. The result will use the aggregate score as the patient's score.

#### 2.5.2 Secondary outcomes

##### 2.5.2.1 Fugl-Meyer movement-function assessment (FMA)

The total score of FMA is 100 points, of which 34 is the total score of lower limb motor function assessment, 66 is the total score of upper limb motor function, and the final score is the sum of the scores of upper and lower limbs. Grade I, severe motor dysfunction (<50 points); Grade II, significant motor dysfunction (50–84 points); Grade III, moderate motor dysfunction (85–95 points); Grade IV, mild motor dysfunction (96–99 points).

##### 2.5.2.2 Modified Barthel index (MBI)

Barthel index is one of the scales widely used at home and abroad to assess the ability of patients to perform activities of daily living. It includes 10 items of basic activities of daily living. In 1989, Shah and Vanchay et al improved the Barthel scale, refined the score, and improved the sensitivity of the scale to changes in patients’ abilities of daily living, thus showing good reliability and validity.^[23]^ The MBI rating scale and scoring criteria were converted into traditional Chinese version by the Department of Rehabilitation Science of The Hong Kong Polytechnic University in the late 1990s. After it was used in Hong Kong hospitals, it was compared with the original English version and revised into simplified Chinese version according to Chinese language habits.^[24]^ The evaluation results of the scale can be divided into 5 levels according to the scores, namely, complete self-care (100 points), basic self-care (75–95 points), need help (50–70 points), partial dependence (25–45 points), and complete dependence (0–20 points).

### 2.6 Statistical methods

#### 2.6.1 Sample size

Our sample size is based on previous studies. In 2014, Lund University, Swedish University of Agricultural Sciences, and southern epidemiology and registration center cooperated in similar experiments, and 101 people were divided into 2 groups to explore the therapeutic effect of natural rehabilitation on PSF. Improvement will be measured according to the same sample size of the estimation formula: n = 2([*μ*_α_+*μ*_β_]^2^*σ*^2^)/*δ*^2^.

With a type I error of 5% (*α* = 0.05) and 90% power (*β* = 0.10), the estimated required sample size is 23 participants per group. Allowing for a 20% dropout rate during the study, a minimum total of 50 participants is needed to reach the target of 23 participants per group.

#### 2.6.2 Statistical analysis

Statistical methods using statistical analysis software SPSS 21.0 (SPSS Inc., Chicago, IL, USA) will be used to analyze the test results. The Shapiro–Wilk test was used to test for normality of data. In the descriptive analysis of samples, the continuous variables are expressed in mean and standard deviation. The normally distributed packets are compared statistically between the *t* test groups. Count data were analyzed using chi-square tests. *P* < .05 was considered statistically significant. Count data were analyzed using chi-square tests. Adverse events will be listed and analyzed using either a chi-square test or Fisher precision test. To test for changes over time, a nonparametric Friedman test was performed for the primary outcome variable FSS. To analyze the effect of real stimulation versus sham stimulation across both primary and secondary outcome measures, the changes in scores were calculated to baseline and then compared using a Wilcoxon signed-rank test.

### 2.7 Randomization and allocation concealment

In this study, randomization and allocation of hidden blocks will be used. Participants will be assigned to either the intervention or control group in a ratio of 1:1. The order of randomization will be derived using the statistical software SPSS24.0 IBM, and performed by an independent statistician from the evidence-based Medicine Center NCU who will not participate in the trial. In addition, randomly assigned ratings will be hidden from the results assessors and statistical analysts. After assessing the basic information of the participants, the allocation of the eligible participants will also be concealed from their care-givers and therapists, including acupuncturists and cognitive therapists, who will be assisting patients to receive treatment.

### 2.8 Blinding

Because of the double-blind implementation of this study, the “third Party” personnel who did not participate in the experiment will manage and supervise the implementation of the blind method:

(1) Patients will not be allowed to open the envelopes indicating the order in which they will be involved in the study. The tDCS model is assumed to be modes A and B, and the project implementer will not know what the stimulus represents.
(2) A mode is the active stimulus, and B is the sham stimulus; treatment outcomes will be assessed by third-party assessors who will not be aware of the grouping.
(3) In order to prevent the analyst's subjective tendency in the process of data analysis, the first non-blind method will be performed before the statistical analysis is completed. In other words, the analyst will know that patients are divided into 2 groups, but will not know which group is the intervention group. After statistical analysis, a second non-blind test will be performed to determine which group will be the intervention group.

## 3 Result

At the end of 12 weeks for treatment and followed-up, the loss rate was 11.67% (7/ 60). The final sample size was 53, including 27 in the control group (n = 27), with a loss rate of 10% (3/30); and 26 in the treatment group (n = 26), with a loss rate of 13.33% (4/30). The specific reasons are as follows: at the 5th week, 1 patient in the control group transferred to another hospital and 29 were retained; at the 7th week, 2 patients in the treatment group dropped, and 1 patient transferred to another hospital, 27 were retained; at the 8th week, 1 patient in the control group dropped, 1 patient transferred to another hospital, 27 were retained, and 1 patient in the treatment group dropped and 26 were retained.

### 3.1 Basic data

Through statistical analysis, there was no significant difference in the basic data of 53 patients between the 2 groups, and it was considered that the difference was not statistically significant (*P* > .05) (Table 2).

**Table 2:** Patient demographics and clinical data for control group and treatment group.

	Control group	Treatment group	*X*^2^/*t*	*P*
Age, yr	59.48 (9.12)	60.42 (9.76)	0.362	.719
Gender (male/female)	18/9	16/10	0.151	.698
Type of stroke ischaemic/hemorrhagic	12/15	12/14	0.016	.901
Hemisphere affected (left/right)	14/13	15/11	0.182	.670
Education years, yr	9.22 (3.32)	8.96 (3.03)	0.297	.768
Time since stroke, d	41.85 (7.41)	42.12 (7.35)	0.133	.895
MMSE scores	21.33 (4.23)	20.88 (4.81)	0.362	.719
FSS scores	46.70 (5.30)	46.04 (5.98)	0.426	.672
FMA scores	46.07 (7.04)	46.19 (6.69)	0.636	.528
MBI scores	71.22 (10.05)	71.88 (9.01)	0.251	.803

### 3.2 Comparison of FSS scores of 2 groups in different time periods

According to the FSS score, a score below 36 indicates that the patient have no longer PSF, while a score above 36 indicates that the patient is still with PSF. Before intervention, patients in both groups had PSF (FSS score ≥ 36). After 8 weeks of intervention, the detection rate of PSF was 70.37% (19/27) in the control group and 38.46% (10/26) in the treatment group. The detection rate in the test group was significantly lower than that in the control group (*P* = .020).

**Figure 2:**
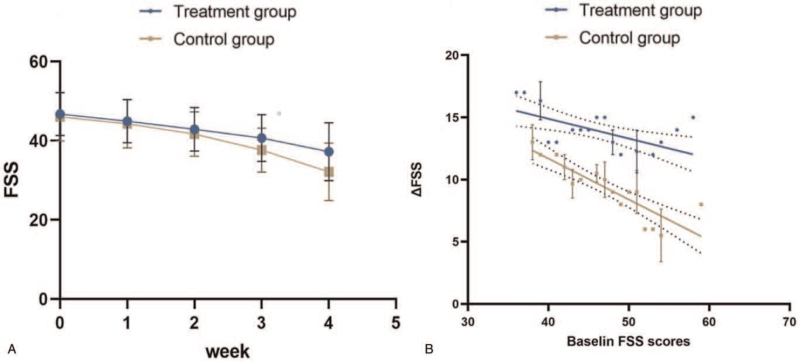
Comparison of BMI scores of 2 groups in different time periods. Comparison of 2 groups of patients in different time periods. A: Comparison of FSS scores between the 2 groups before and after intervention. B: Comparison of FSS scores between the 2 groups before and after followed up. C: Comparison of FMA scores of 2 groups of patients in different time periods. D: Comparison of MBI scores of 2 groups of patients in different time periods. FAM = Fugl-Meyer movement-function assessment, FSS = fatigue severity scale.

According to the test, there was no significant difference in the score of FSS between the 2 groups before intervention (*P* = .838). The scores of the 2 groups after intervention are both decreased, and the differences were statistically significant (*P* < .001). After 4 weeks of intervention, the score of the control group was significantly higher than that of the treatment group. According to the test, the difference between the 2 groups was statistically significant (*P* = .012) (Fig. 2A) (Table 3).

**Table 3:** Comparison of FSS scores of 2 groups before and after treatment.

	Before treatment	After treatment	*t/Z*	*P*
Control group	46.70 ± 5.30	37.22 ± 7.17	5.525	<.001
Treatment group	46.38 ± 5.98	32.12 ± 7.10	7.833	<.001
*t/Z*	0.206	2.601		
*P*	.838	.012		

At the end of the 8-week follow-up, FSS scores were collected again and compared with those before the follow-up. By calculation, there was no significant difference in FSS scores between the experimental group and the control group before and after follow-up (*P* > .05). After follow-up, the FSS score of the control group was still significantly higher than that of the experimental group (*P* < .001). This indicates that the effect of tDCS on improving PSF is sustainable and is not easy to relapse (Fig. 2B) (Table 4).

**Table 4:** Comparison of FSS scores of 2 groups before and after follow-up.

	Before follow-up	After follow-up	*t/Z*	*P*
Control group	37.22 ± 7.17	38.09 ± 7.16	0.446	.657
Treatment group	32.12 ± 7.10	31.35 ± 5.47	0.438	.663
*t/Z*	2.601	3.814		
*P*	.012	<.001		

**Figure 3:**
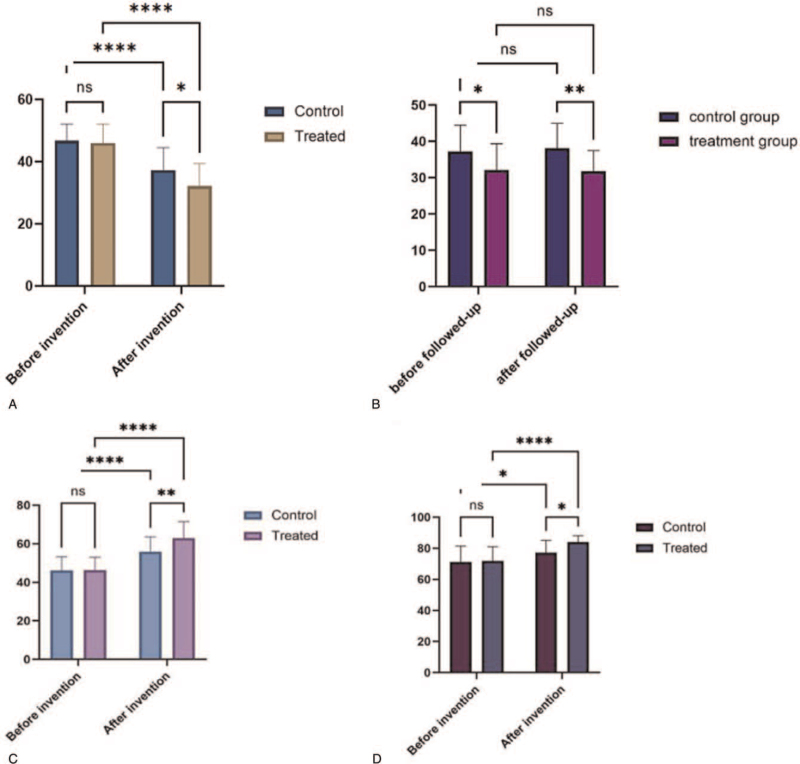
Comparison of FSS scores of 2 groups in different time periods. A: FSS scores in different weekend. B: Correlation between baseline FSS and the change in FSS. FSS = fatigue severity scale.

In the course of treatment, with the accumulation of treatment times, the treatment effect is more significant (Fig. 3A). The higher the baseline FSS is, the greater the decrease in FSS is, and the more significant the treatment effect is (Fig. 3B).

### 3.3 Comparison of FMA scores of 2 groups in different time periods

Before intervention, there was no significant difference in FMA index between the 2 groups by *t* test (*P* = .949). The FMA index of the 2 groups increased a lot after intervention (*P* < .001), and the *t* test indicated that there were significant differences between the index before and after the intervention. After 4 weeks of intervention, the score of the control group was significantly lower than that of the experimental group (*P* = .003), and the difference was statistically significant (Fig. 2C) (Table 5).

**Table 5:** Comparison of FMA scores of 2 groups before and after treatment.

	Before treatment	After treatment	*t/Z*	*P*
Control group	46.07 ± 7.04	55.93 ± 7.50	4.937	<.001
Treatment group	46.19 ± 6.69	62.96 ± 8.47	8.015	<.001
*t/Z*	0.064	3.168		
*P*	.949	.003		

### 3.4 Comparison of Body Mass Index (BMI) scores of 2 groups in different time periods

Before intervention, there was no significant difference in MBI index between the 2 groups by *t* test (*P* = .801). After 4 weeks of intervention, the MBI index of control group was significantly higher that before intervention (*P* = .019). It's the same with the treatment group with more significant difference (*P* < .001). After 8 weeks of intervention, the score of the control group was significantly lower than that of the experimental group (*P* < .001), indicating that there was a significant difference between the 2 groups (Fig. 2D) (Table 6).

**Table 6:** Comparison of BMI scores of 2 groups before and after treatment.

	Before treatment	After treatment	*t/Z*	*P*
Control group	71.22 ± 10.05	77.22 ± 7.81	2.420	.019
Treatment group	71.88 ± 9.014	83.96 ± 4.11	6.236	<.001
*t/Z*	0.254	3.894		
*P*	.801	<.001		

### 3.5 Adverse reactions

**Figure 4:**
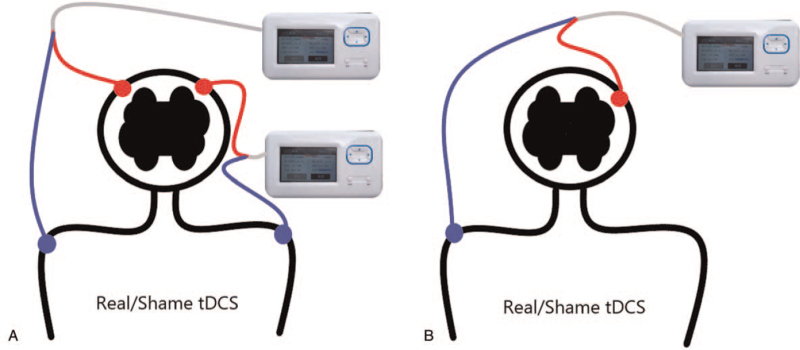
The difference in the site of stimulation. A: Stimulation method in study by Doncker et al^[46]^ at January 21, 2021. B: Stimulation method in study described above.

Through the whole trial, the adverse reactions of patients in the trial group were as follows: 53.85% (14/26) of patients had mild tingling and 7.69% (2/26) had mild itching, which were acceptable and would not cause excessive discomfort. No adverse reactions such as burns and nausea occurred. According to the examinations during the trial, the vital signs of the patients were stable and did not fluctuate too much, suggesting that it is safe with appropriate intensity, fault-free equipment, and standard therapists’ operating (Fig. 4).

## 4 Discussion

As a simple, low-cost and non-invasive technology, tDCS has gradually entered the vision of public in recent years. Composed of 2 surface electrodes, cathode and anode, it acts on the cerebral cortex with weak direct current passing through the surface of the brain. There have been more and more studies about tDCS in recent years, including reports on improving fatigue by affecting the central nervous system.^[12,25–28]^ This study has achieved significant experimental results in exploring the effect and safety of routine treatment combined with tDCS in improving PSF. Through the study, the rate of loss was low, and no patient dropped from the experiment because of serious unbearable adverse reactions, which suggested that the compliance and the safety of tDCS treatment. With the development of the society, the survivals of stroke have increased significantly, and the sequela of stroke has become one of the main reasons affecting people's health level. As a common complication after stroke, PSF has gradually been known and paid attention to by public, attracting the attention of the society.^[29]^ Some studies have shown that PSF has been one of the independent risk factors for the increase of mortality and the decrease of long-term survival rate in patients after stroke.^[30]^

The pathogenesis of PSF is not clear. Studies have shown that fatigue in patients may involve central and peripheral abnormalities, and central changes may play a major role.^[31]^ Central fatigue may be caused by 3 main neurophysiological mechanisms: changes in the synthesis and metabolism of neurotransmitters in the brain,^[32–34]^ decreased discharge frequency of spontaneous neurons,^[35]^ and damage of cortical areas associated with movement.^[36,37]^

The central fatigue neurotransmitter hypothesis holds that long-term activity could lead to the change of the synthesis and metabolism of monoamine neurotransmitters in the brain, such as 5-hydroxytryptamine (5-HT), dopamine (DA), and γ-aminobutyric acid (GABA), resulting in increased sleep and loss of drive. This is why people feel tired. The mechanism of tDCS to improve fatigue may be related to the regulation of neurotransmitter synthesis and metabolism in the brain. Studies^[38–40]^ found that tDCS can significantly increase the levels of DA and 5-HT in the frontal cortex and improve the depressive behavior of rats. What's more, the effect of anode is more obvious. And the mechanism may be related to the increase of monoamine neurotransmitters in related brain regions. Other studies^[41]^ have shown that tDCS can also decrease the activity of GABA, increase the level of intracellular Ca^2+^ and change the level of brain-derived neurotrophic factor (BDNF). This may be one of the ways tDCS works in PSF.

The study of Bruno et al^[33]^ shows that the change of spontaneous discharge of neurons may be one of the important causes of persistent fatigue in patients with stroke. Ciechanski et al^[42]^ found that tDCS could enhance neuronal connections with a long-term enhancement mechanism by increasing neuronal spontaneous discharge and synaptic efficiency. However, recent study^[43]^ have shown that under the stimulation intensity commonly used in the study, tDCS had no systematic effect on discharge rate or discharge pattern. Subtle but statistically insignificant changes occur only in sites with the most sensitivity or the highest intensity of anodic stimulation, which suggested that the effects of tDCS observed in previous study were likely to occur through mechanisms other than direct neuronal depolarization. When studying the spontaneous discharge of globus pallidus neurons, study^[44]^ found that 5-HT could enhance the spontaneous discharge of neurons. Combined with what was mentioned that tDCS could regulate the synthesis and metabolism of neurotransmitters, it is speculated that its mechanism may be to change the spontaneous discharge of neurons by affecting neurotransmitters. This could explain why tDCS is effective in alleviating PSF.

A number of studies^[33]^ have shown that central fatigue may depend on low activation in the frontal lobe decreased sensitivity to stimuli, and delayed or disappeared response to the same stimuli. It is speculated that transcranial direct current stimulation can significantly improve the fatigue of patients by restoring the excitation ability of neurons and axons in the prefrontal region. Studies by Cole et al^[45]^ have shown that tDCS can regulate the membrane potential of neurons through DC subthreshold stimulation, change the excitability of cortical neurons, and repair brain injury, thus improving brain function. The effective mechanism may be that the application of micro-current changes the voltage difference between inside and outside the neuron membrane, and then changes the neuron discharge threshold. Cortical neurons are sensitive to DC electric field, which leads to changes in the operation of Na^+^-K^+^ pump and local transmembrane ion concentration. When the current anode was used to stimulate the neuron, the discharge of the neuron increased, and the excitability was enhanced. The excitability and functional characteristics of prefrontal cortex neurons can be regulated by using transcranial DC anode to stimulate the appropriate body surface brain area.^[32]^

A recent study^[46]^ found that a single session of anodal tDCS improves fatigue symptoms with the effect lasting up to a week post stimulation. However, there are many differences from this study (Table 7). As showed in the table, the 2 experiments have different focuses of exploration. The study by Doncker et al^[46]^ explore the effect of once stimulation, while this study explores the effect of continuous treatment for weeks. Whatever, we all found tDCS to be effective in the treatment of PSF.

**Table 7:** Compare the differences between the 2 experiments.

	Trial.1^∗^	Trial.2^†^
Inclusion criteria		
Time after stroke	>3 months	>21 days
First stroke	Yes	No requirement
Sample size (control group/treatment group)	30 (10/20)	53 (27/26)
Stimulation method (cathode/anode)		
Number	2/2	1/1
Site	Shoulders/M1 (left and right)	DLPFC/Superior margin of right orbit
Magnitude of current	2 mA	1.5 mA
Electrode		
Shape	Square	Circle
Size	5 cm × 7 cm	d = 5cm
Stimulation time	1 time	24 times (in 4 weeks)
Outcomes		
Primary outcome	FSS-7	FSS
secondary outcomes	PE	FMA
	MEP	MBI

### 4.1 Limitations and outlook

This is a double-blind randomized controlled trial whose aim is to assess the effects of tDCS on PSF. With a period of follow-up, this study can provide more information about the treatment of PSF. However, our research has some limitations. Firstly, the related scale used to evaluate the fatigue degree of patients is greatly affected by the subjective factors of patients. Therefore, the experimental results are easily affected by various factors, such as the favorite degree of therapists. The FSS has good internal consistency, reliability, construct and criterion validity, and is sensitive to change. It has been evaluated in several conditions where it is the recommended fatigue scale.^[47,48]^ Thus, we can maximize the accuracy of the experiment. Secondly, because it is a single-center trial and a relatively small number of participants, the influence of chance on experimental results cannot be completely ruled out. It forms an integral aspect of our future research work to provide a new medical basis for the treatment of patients with tDCS. Thirdly, all of our outcome evaluations, including primary and secondary outcomes, were scale evaluations, lacking measurement of objective indicators, which will provide some inspiration for subsequent experiments.

Because of the high incidence of post-stroke fatigue, which has a great impact on the life of patients, it is particularly important to explore an effective way to treat PSF. This experiment on the therapeutic effect of tDCS on PSF will provide a new direction. This experiment is an active exploration for the treatment of PSF.

In summary, we found that tDCS at 1.5 mA to DLPFC is effective for treatment of PSF. And it's work can last for a long time. This suggests tDCS has great potential for PSF, in combination with conventional rehabilitation.

## Acknowledgments

The authors thank the rehabilitation department of The First Affiliated Hospital of Nanchang University, and all patients who participated in the study.

## Author contributions

**Conceptualization:** Weiming Sun.

**Data curation:** Xing Sun.

**Formal analysis:** Xiangli Dong.

**Funding acquisition:** Xiangli Dong, Weiming Sun.

**Investigation:** Xiangli Dong.

**Methodology:** Guohua Yu.

**Project administration:** Guohua Yu.

**Resources:** Guohua Yu.

**Software:** Qin Yuan, Lang Shuai.

**Supervision:** Qin Yuan, Lang Shuai.

**Validation:** Qin Yuan, Lang Shuai.

**Visualization:** Yefeng Yuan.

**Writing – original draft:** Xing Sun, Weiming Sun, Yefeng Yuan.

**Writing – review & editing:** Xing Sun, Weiming Sun, Yefeng Yuan.
